# Enabling tomography with low-cost C-arm systems

**DOI:** 10.1371/journal.pone.0203817

**Published:** 2018-09-13

**Authors:** Monica Abella, Claudia de Molina, Nerea Ballesteros, Alba García-Santos, Álvaro Martínez, Inés García, Manuel Desco

**Affiliations:** 1 Dept. Bioingeniería e Ingeniería Aeroespacial, Universidad Carlos III de Madrid, Madrid, Spain; 2 Instituto de Investigación Sanitaria Gregorio Marañón, Madrid, Spain; 3 Centro Nacional Investigaciones Cardiovasculares Carlos III (CNIC), Madrid, Spain; 4 Centro de investigación en red en salud mental (CIBERSAM), Madrid, Spain; North Shore Long Island Jewish Health System, UNITED STATES

## Abstract

In scenarios where the use of a Computed Tomography (CT) is difficult, such as during surgery or in the ICU, the use of a C-arm system to generate tomographic information could contribute with interesting additional clinical information. Recent days are seeing the development of the so-called cone-beam CT (CBCT) based on advanced motorized isocentric C-arm systems. To be able to make use of more basic C-arm systems, apart from the geometric non-idealities common to any CBCT, we need to address other difficulties. First, the trajectory of the source-detector pair may differ from a circular path and the system may suffer mechanical strains that modify the relative positions of the source and detector for different projection angles. Second, and more importantly, the exact position of the source and detector elements may not be repeatable for consecutive rotations due to low mechanical precision, thus preventing an accurate geometrical calibration of the system. Finally, the limitation of the angular span and the difficulty of obtaining a high number of projections pose a great challenge to the image reconstruction. In this work, we present a novel method to adapt a standard C-arm, originally designed for planar imaging, to be used as a tomograph. The key parts of the new acquisition protocol are (1) a geometrical calibration method to compensate mechanical inaccuracies that prevent an accurate repetition of source-detector position between acquisitions, and (2) an advanced image reconstruction method able to deal with limited angle data, sparse projections and non-circular trajectories. Both methods exploit surface information from the patient, which can be obtained using a 3D surface scanner. The proposed method was evaluated with two real C-arm systems, based on an image intensifier and a flat panel detector respectively, showing the feasibility of the proposal.

## Introduction

A C-arm is a fluoroscopic system comprising two units, an X-ray generator and a detector (image intensifier or flat panel) mounted in an arc-shaped gantry, together with a workstation used to visualize, store, and manipulate the images. Designed to acquire real-time planar images, C-arms have demonstrated to be a useful qualitative assessment tool to guide surgical procedures thanks to their open design, compactness and portability, which allow to set the C-shape around the patient lying in the bed [1]. Another advantage is their low cost in comparison with other medical image modalities. However, there is an important drawback intrinsic to its 2D nature: the lack of depth information.

In situations where a Computed Tomography (CT) system is not an option (either because unavailability or patient mobility restrictions), the use of a C-arm as a tomograph would raise the possibility of generating tomographic information, with the potential of improving diagnosis and likely surgical precision [2]. So-called cone-beam CTs, based on advanced isocentric motorized C-arm systems (generally attached to a gantry), have been used in a broad range of image-guided procedures, including image-guided radiotherapy [3–5], mammography [6], dentistry imaging [7, 8] and general surgical procedures [9–11].

When cost is a relevant issue, it could be helpful to obtain tomographic information using more basic C-arm systems. The straightforward use of a standard non-isocentric C-arm for tomography presents significant difficulties, apart from the geometrical non-idealities common to any Cone Beam Computed Tomography (CBCT). The first important problem to face is that the relative positions between source and detector may change for different projections. This derives from the mechanical strains in the arm due to the heavy loads at its ends. This fact, together with the non-circular orbit of C-arms determined by the slipping of the arm over the base, hinders the use of calibration methods widely used in CT systems that obtain global calibration parameters for all projections, such as the one proposed in [12]. Other different methods have been proposed to generate the geometrical calibration of an imaging system projection by projection. The approach based on the so called “camera model” calibration, used for planar imaging, has shown instability [6] and does not provide the center of rotation, rendering it not suitable for tomography systems. Cho et al. [13] proposed a method specifically designed to obtain calibration parameters of a cone-beam CT system individually for each projection, based on the acquisition of a simple phantom with two circular patterns.

A second problem is that the utility of the geometrical parameters obtained with any periodical calibration procedure depends on the mechanical stability of the imaging system. For C-arms aimed for planar imaging, the exact position of the source and detector elements tends not be repeatable for consecutive rotations due to low mechanical precision, thus making it impossible to achieve the accuracy required for obtaining good quality 3D reconstructions.

Finally, due to physical movement limitations, only a few projections can be acquired, typically covering a much smaller angular span than the one used in conventional CBCT, where the system rotates around the patient through 360 degrees (full angular span), generally acquiring more than 360 projections. The reconstruction of these limited data implies an extremely ill-posed inverse problem where the use of conventional methods, such as the one proposed by Feldkamp, Davis and Kress (FDK) [14], results in severe artifacts in the image (streaks and/or shape distortion).

In this work, we present a method based on the use of patient surface information obtained with a 3D surface scanner that enables adapting a standard C-arm, originally designed for planar imaging, to be used as a tomograph. The key parts of the novel method are a geometrical calibration that compensates the high mechanical inaccuracy that prevent any accurate repetition of source-detector positions between acquisitions together with an advanced image reconstruction algorithm able to deal with very limited angular span and non-circular trajectories.

## Proposed calibration-reconstruction protocol

**Fig 1:**
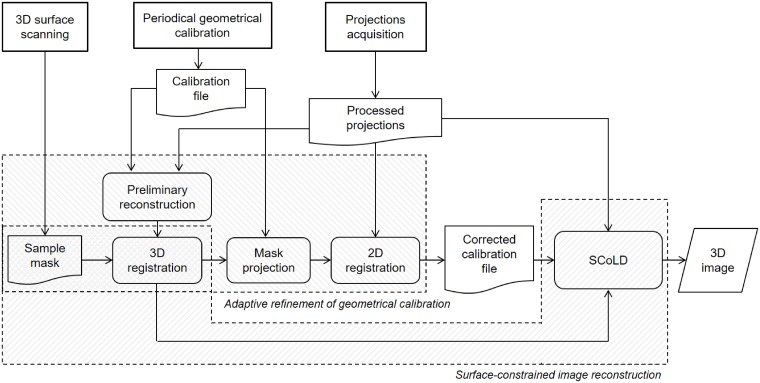
Workflow of the acquisition/reconstruction process.

Fig 1 shows the workflow of the proposed method.

**Fig 2:**
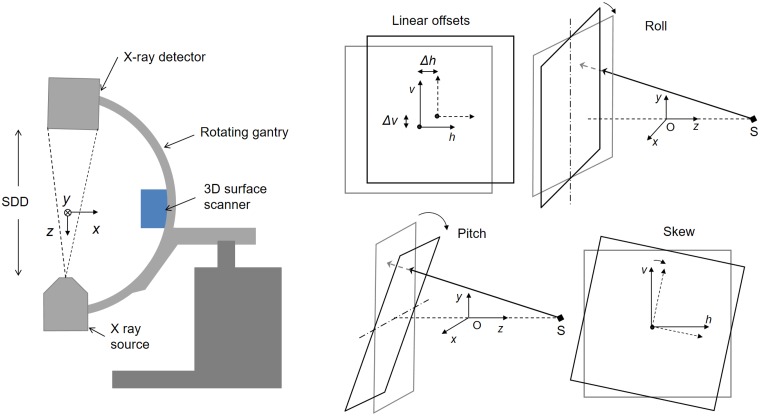
Scheme depicting the coordinate system and the geometrical misalignments in the detector panel.

A geometrical calibration of the system is obtained periodically, as it is customary in standard CT scanners, with an algorithm based on a method specifically designed to obtain individual calibration parameters for each projection angle proposed by Cho et al. [13]. The result of the calibration is the set of parameters values shown in the right panel of Fig 2 for each projection angle: detector rotation (skew), inclination angles (pitch and roll), piercing point location (projection of the center of the calibration phantom), SDD (source to detector distance) and source and detector position.

For each study, together with the projection data acquired at different source-detector positions we also obtain the surface information of the sample using a 3D surface scanner. We obtain a preliminary reconstruction of the sample from the projection data with the FDK-based method proposed in [15] using the geometric calibration data. In parallel, we obtain a binary mask of the sample, and perform a 3D rigid registration based on mutual information with the preliminary reconstruction.

The generation of the tomographic image is then achieved in two steps, using the previously registered mask of the sample. First, we refine the system calibration to calculate accurate positions of the source and detector in the current acquisition, by comparing the projections of the registered mask with the acquired projection data. Second, using the refined calibration, the image is reconstructed from the projection data also taking advantage of the available registered mask of the sample.

The key steps of the proposed method are detailed in the following sections.

### Geometric calibration

The algorithm we use for the geometric calibration is based on the work by Cho et al [13]. As a thorough description can be found in the paper by Cho et al [13], in this section we only describe the key points and details specific for our implementation.

The calibration phantom is a Polymethyl methacrylate cylinder with two circular patterns formed by ball bearings symmetrically embedded in the cylinder wall. Projections of the phantom are obtained at the angular positions expected to be used in the specific trajectory during the acquisition of the sample. For each projection, the algorithm completes 6 steps: 1) identification of center of mass positions of ball bearings, 2) ellipse fitting, 3) determination of *v*-*h* offset, 4) determination of skew angle (*η*), 5) determination of converging point and inclination angles (*θ* and *φ*), and 6) determination of source and detector positions.

**Fig 3:**
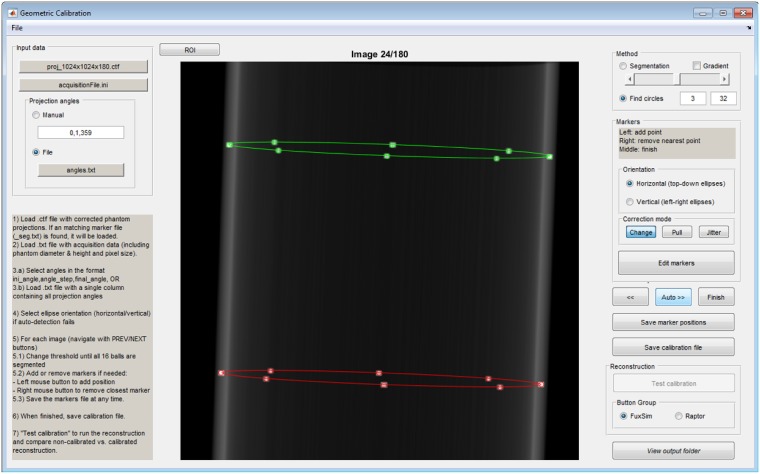
System geometric calibration tool showing the ellipses for one projection of the calibration phantom.

An initial segmentation of the markers is achieved in a semi-automatic way using a calibration program with an interactive graphic user interface (Fig 3), based on a global thresholding segmentation followed by morphological operations to remove artifacts. An initial threshold is estimated by Otsu’s method [16], but can be interactively adjusted by the user with a slider. Once the segmented markers are well identified, they are automatically classified into two sets (corresponding to each ring), according to their relative position with respect to the image center. To overcome the problem of overlapping markers, the tool allows the user to interactively correct the marker positions (place, remove or reposition), showing the updated ellipses overlaid to the image in real time.

Following Cho et al [13], ellipses are parametrized as:

$$a{\left(h-h_{0}\right)}^{2}+b{\left(v-v_{0}\right)}^{2}+2c(h-h_{0})(v-v_{0})=1$$ (1)

where (*h*_*0*_, *v*_*0*_) are the coordinates of the ellipse center and *a*, *b*, and *c* are parameters that describe its shape. To relate these parameters with the coordinates of the centers of mass of the balls previously obtained with the calibration program (*h*_*b*_, *v*_*b*_), we use the polynomial description of the ellipse:

$$p_{0}h_{b}^{2}+v_{b}^{2}-2p_{1}h_{b}-2p_{2}v_{b}+2p_{3}h_{b}v_{b}+p_{4}=0$$ (2)

where *p*_*i*_ are obtained solving the following system:

$$\left(\begin{array}{ccccc} h_{0}^{2} & -2h_{0} & -2v_{0} & 2h_{0}v_{0} & 1\\ & & \vdots & & \\ h_{7}^{2} & -2h_{7} & -2v_{7} & 2h_{7}v_{7} & 1 \end{array}\right)\left(\begin{array}{c} p_{0}\\ \vdots \\ p_{7} \end{array}\right)=\left(\begin{array}{c} -v_{0}^{2}\\ \vdots \\ -v_{7}^{2} \end{array}\right)$$ (3)

Finally, we calculate ellipse parameters in Eq (1) using the relations described by Noo et al. [12]:

$$h_{0}=\frac{\left(p_{1}-p_{2}p_{3}\right)}{\left(p_{0}-p_{3}^{2}\right)},{\text{v}}_{0}=\frac{\left(p_{0}p_{2}-p_{1}p_{3}\right)}{\left(p_{0}-p_{3}^{2}\right)},a=\frac{p_{0}}{\left(p_{0}h_{0}^{2}+v_{0}^{2}+2p_{3}h_{0}v_{0}-p_{4}\right)},b=\frac{a}{p_{0}},c=p_{3}b$$ (4)

**Fig 4:**
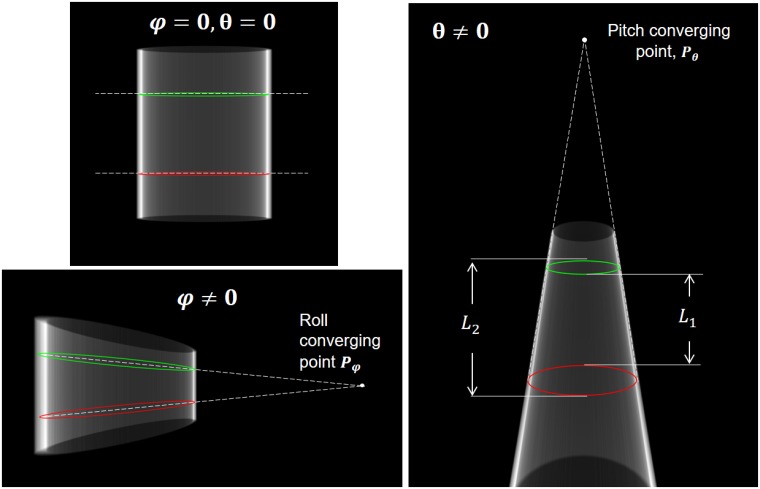
Phantom projection for the case with no detector panel inclinations (top-left), for roll angle *φ* = 60 degrees (bottom-left) and for pitch angle *θ* = 60 degrees (right).

We then calculate the horizontal and vertical displacements and the skew of the detector panel, as well as the source and detector positions following the Cho et al. method [13]. Regarding inclination angles, if both pitch angle, *θ*, and roll, *φ*, are zero, both ellipses have the same shape and their long axis are parallel, as shown in Fig 4 (top, left). When *θ* is different from zero, one ellipse is lengthier than the other, as illustrated in the right panel of Fig 4. The lines tangent to both ellipses converge to the *pitch converging point*, *P*_*θ*_.

Since the calculation of both inclination angles in Cho et al. is based on *P*_*θ*_ (equation 18 in [13]) pitch angle *θ* is assumed to be different from zero. To remove this restriction, we propose the following modification to the Cho’s method to estimate both effects separatelly.

The pitch angle is obtained from the geometric relationships:

$$\theta =\text{arcsin}\left[\frac{Z_{S}\text{cos}(\varphi )}{v_{\theta }}\right],Z_{S}=\left[\frac{2\cdot R\cdot L_{1}\cdot L_{2}}{H\cdot \left(L_{2}-L_{1}\right)}\right]$$ (5)

where *Z*_*S*_ is the source distance, *L*_*1*_ and *L*_*2*_ are the distances between the two ellipses (Fig 4, right), and *R* and *H* are the radius and the distance between the two circular patterns in the real phantom.

The roll angle is calculated by considering that when *φ* is different from zero, the long axis of both ellipses converge to the roll converging point *P*_*φ*_(*h*_*φ*_, *v*_*φ*_), as shown in the left-bottom panel of Fig 4. Roll angle, *φ*, is obtained using the Nelder-Mead simplex method [17] to minimize the cost function *C*_*φ*_:

$$C_{\varphi }=\text{sin}(\varphi )+{\text{c}}_{1}\cdot {ℶ}_{1}/2\cdot (a_{1})+{\text{c}}_{2}\cdot {ℶ}_{2}/2\cdot (a_{2})$$ (6)

where *ℶ*_*1*_ and *ℶ*_*2*_ are intermediate parameters for each ellipse given by:

ℶk=T⋅a⋅kakak⋅bk+ak2⋅bk⋅(ZS′)2−ck2,k=1,2 (7)

with *T* given by

$$T=h_{\varphi }\cdot \text{sin}(\varphi )\cdot \text{cos}(\varphi )$$ (8)

### Adaptive refinement of geometrical calibration

**Fig 5:**
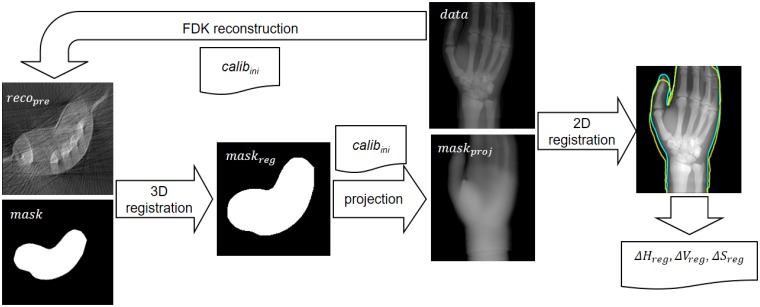
Workflow of the adaptive refinement of geometrical calibration algorithm. Yellow and blue lines show the contour of the projected mask and the projection data respectively.

The first algorithm of our proposed method, *adaptive refinement of geometrical calibration*, tackles the problem of low repeatability of the exact positions of the source and detector due to the high mechanical inaccuracy of these systems. This is achieved through the refinement of geometrical parameters in the detector plane: horizontal offset, *Δh*, vertical offset, *Δv*, and skew, *ΔS* (Fig 2). Fig 5 shows a graphical workflow of the algorithm.

First, we generate a preliminary reconstruction, *reco*_*pre*_, which is used to orientate and adjust the mask to the field of view of the C-arm, by means of a 3D registration based on mutual information. The registered mask is then projected using the initial system calibration, *calib*_*ini*_. The misalignment between the projections of the mask, *mask*_*proj*_, and the acquired data, *data*, reflect the errors in the values *H*, *V*, and *S* of *calib*_*ini*_. Therefore, the parameters of a 2D registration between *mask*_*proj*_ and *data* at each projection angle, *ΔH*_*reg*_, *ΔV*_*reg*_ and *ΔS*_*reg*_, are used to generate refined values *U*_*corr*_, *V*_*corr*_, and *S*_*corr*_ as:

$$H_{corr}=H+\Delta H_{reg}+O_{h}-\left(O_{h}\cdot \text{cos}\left(\Delta S_{reg}\right)-O_{v}\cdot \text{sin}\left(\Delta S_{reg}\right)\right)$$ (9)

$$V_{corr}=V+\Delta V_{reg}+O_{v}-\left(O_{h}\cdot \text{sin}\left(\Delta S_{reg}\right)+O_{v}\cdot \text{cos}\left(\Delta S_{reg}\right)\right)$$ (10)

$$S_{corr}=S-\Delta S_{reg}$$ (11)

where *O*_*h*_ and *O*_*v*_ are the coordinates of the detector center.

### Surface-constrained image reconstruction

Image reconstruction of the limited data is done with a Surface-Constrained Method for Limited Data tomography (SCoLD) developed by our group [18], using projection-backprojection kernels implemented in GPU to reduce computational burden [19].

The reconstruction algorithm follows the idea of the Total Variation (TV) minimization subject to a support constraint, which contains the *a priori* sample mask information, formulated as:

$$\underset{u}{\text{min}}TV(u),s.t.{\left\|Au-f\right\|}_{2}^{2}<\sigma^{2},u\geq 0,u\in \Omega$$ (12)

where *u* is the reconstructed image, *Ω* the subspace that corresponds with the surface support of the sample, *A* is the system matrix, *f* represents the acquired data and *σ*^2^ is the image noise. The L_1_-constrained optimization problem, shown in Eq (12), is efficiently solved using a Split Bregman formulation [20] and expressed as the following unconstrained problems, which are sequentially solved at each iteration *k*:

$$\begin{array}{r} \left(u^{k+1},d_{x}{}^{k+1},d_{y}{}^{k+1}\right)=\underset{u,d}{\text{min}}{\left\|\left(d_{x},d_{y}\right)\right\|}_{1}+\frac{\mu }{2}{\left\|Au-f^{k}\right\|}_{2}^{2}+\frac{\lambda }{2}{\left\|d_{x}^{k}-\nabla_{x}u-b_{x}^{k}\right\|}_{2}^{2}+\\ +\frac{\lambda }{2}{\left\|d_{y}^{k}-\nabla_{y}u-b_{y}^{k}\right\|}_{2}^{2}+\frac{\gamma }{2}\left\|v-u-b_{v}^{k}\right\| \end{array}$$ (13)

$$b_{x}^{k+1}=b_{x}^{k}+\nabla_{x}u^{k+1}-d_{x}^{k+1},b_{y}^{k+1}=b_{y}^{k}+\nabla_{y}u^{k+1}-d_{y}^{k+1}$$ (14)

$$f^{k+1}=f^{k}+f-Au^{k+1}$$ (15)

$$b_{v}^{k+1}=b_{v}^{k}+u^{k+1}-v^{k+1}$$ (16)

Eq (13) leads to two sub-problems: the first one, which contains only L_2_ norm terms, is solved iteratively using a Krylov space solver; the second one, with the L_1_ terms, is solved using analytical formulas. Eqs (14), (15) and (16) are the Bregman iterations that impose the data constraint and surface constraint, respectively.

## Evaluation with real systems

**Fig 6:**
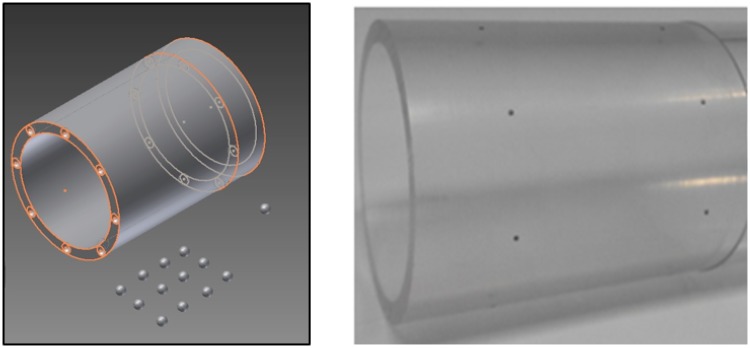
Design of the calibration phantom (left) and real phantom (right).

We have tested the proposed method using two different C-arm devices: a commercial C-arm based on an image intensifier (SIREMOBIL, SIEMENS) and an in-house C-arm prototype based on a flat panel detector. The calibration was performed for each system using a calibration phantom with two circular patterns of diameter 49.1 mm separated 35 mm, each one formed by eight ball bearings (with 0.8 mm of diameter) symmetrically located around a cylinder with internal and external diameters of 45.5 mm and 49.5 mm respectively (Fig 6).

For testing we used one hand and one foot of a PBU-60 anthropomorphic phantom (Kyoto Kagaku Co., Kyoto, Japan). Both phantoms were acquired beforehand in a Toshiba Aquilion/LB helical scanner and reconstructed as a CT volume of 512×512×1645 voxels, with 0.931×0.931×0.5 mm voxel size. A simulated mask was obtained from the CT volume by thresholding.

**Fig 7:**
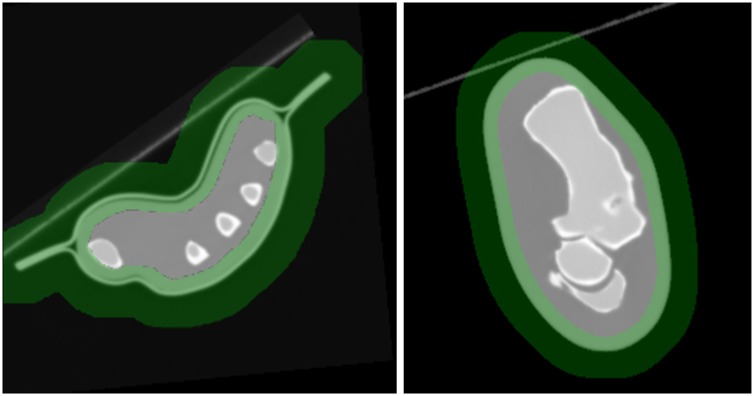
Central axial slice of the CT volume corresponding to the hand (left) and the foot (right) phantoms with the ROI used for the calculation of LiVA overimposed in green.

Quantitative evaluation was done using two metrics. The reduction of the artifacts due to the low number of projections was evaluated using the Streak Artifact Indicator (SAI), which measures the total variation of the difference between reconstructed images and the CT volume [21]. The compensation of the distortion due to the limited angular span was measured with the Limited View Artifact metric (LiVA). LiVA was calculated as the RMSE between the reconstructed images and the CT volume within an ROI delimited by the external contour of the sample, as shown in Fig 7 for the two phantoms used.

Data were reconstructed on a computer with an Intel(R) Core(TM) i7- 7700 processor at 3.6 GHz and one NVidia GeForce GTX 1060 6GB GPU with the FDK-based method proposed in [15] and SCoLD with *μ* = 35, *λ* = 5, *γ* = 0.02 and 35 iterations.

### C-arm Model SIREMOBIL

**Fig 8:**
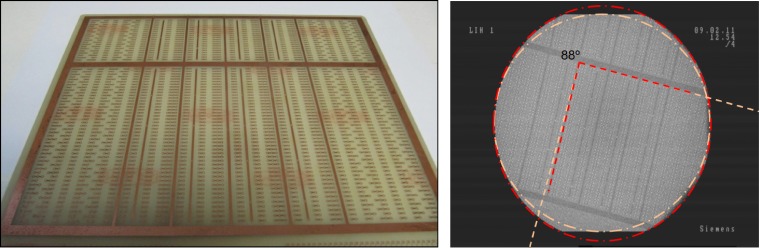
Left: Electronic board consisting of a cupper layer on a plastic plate used to evaluate distortions in the image intensifier. Right: Phantom projection acquired with the C-arm; red lines follow the obtained pattern and yellow lines show the ideal directions and shapes of the phantom.

The C-arm Model SIREMOBIL Compact L of Siemens consists of an X-ray source and a 23 cm-diameter image intensifier attached to a C-arm gantry. The detector of the SIREMOBIL system is an X-ray image intensifier (XRII) connected to a TFT monitor for image visualization. We exported the acquired images to a PC through a USB port using an external video-capture device (model NPG USB RealStudio II), which copied static images and video data onto the connected computer. To evaluate the lines distortion and vignetting of the intensifier, we used a phantom consisting of an old electronic board with radio-opaque cupper straight lines at right angles (Fig 8, left) attached to the intensifier outer casing. Profiles taken along the line patterns on the projection images showed a straight pattern indicating no significant line distortion or vignetting in the image intensifier. Nevertheless, the projection showed a slightly oval shape (yellow lines in Fig 8, right) that does not match the circular shape of the image intensifier case.

A difference of 8% found between small and large diameter was corrected by the geometrical transformation:

$$\left(\begin{array}{l} x´\\ y´\\ 1 \end{array}\right)=\left(\begin{array}{ccc} 1 & 0 & 0\\ 0 & 1.08 & 0\\ 0 & 0 & 1 \end{array}\right)\left(\begin{array}{l} x\\ y\\ 1 \end{array}\right)$$ (17)

**Fig 9:**
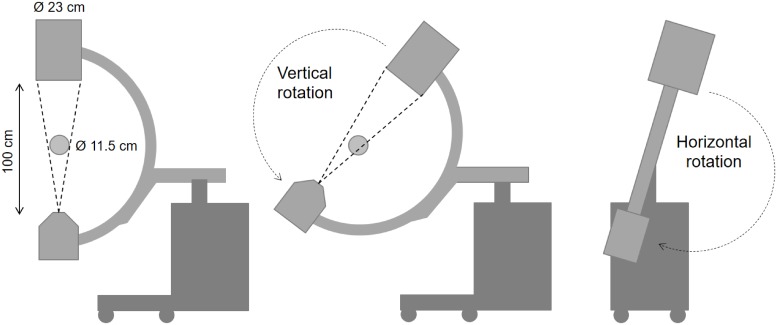
SIREMOBIL geometry (left), non-isocentric vertical rotation movement showing the dimensions of detector and FOV (center), and horizontal rotation movement (right).

The rotation around *y* axis (vertical rotation in Fig 9) is severely non-isocentric due to the slipping of the arm over the base. To maintain a reasonable Field of View (FOV) size seen from all projection angles, we acquired 60 projections rotating around the horizontal supporting arm (horizontal rotation in Fig 9), following an orbit close to be isocentric.

**Fig 10:**
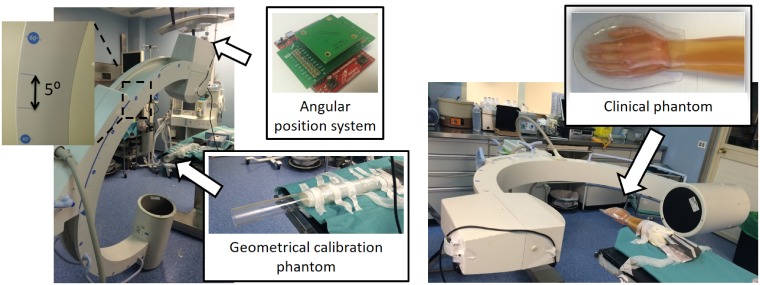
Setting for calibration phantom (left) and clinical phantom (right) acquisitions, showing the angular position recording system.

As with other conventional C-arms, not designed for tomography, the movement of our device is manual and the only angular positioning information provided is a rudimentary scale drawn on the arm (Fig 10, left). To increase the accuracy of angular positioning, we implemented a position-recording device based on an ADIS16209 digital inclinometer, with an accuracy of 0.1 degrees (Fig 10, left). This system was connected to the computer by a single-board microcontroller, the model TM4C123G of LaunchPad board (Texas Instruments). To avoid inaccuracies due to possible vibration of the C-arm, we averaged ten consecutive readings from the inclinometer. Position values, measured as relative increments in the range of ±90 degrees, were transformed into absolute values ranging from 0 to 360 degrees to be used in the reconstruction. The direction of rotation was estimated from previous recorded positions.

**Fig 11:**
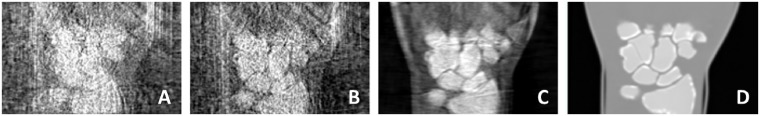
Axial and coronal views of the reconstruction of the PBU-60 phantom. A and B correspond to FDK before and after adaptive calibration respectively. C is the reconstruction with SCoLD and D is the CT volume acquired on the helical scanner.

Fig 11 shows axial, sagittal and coronal views of the image reconstructed with FDK (before and after *adaptive refinement of geometrical calibration*) and reconstructed with SCoLD.

### Evaluation with in-house built C-arm prototype

The in-house built C-arm prototype is based on a wireless, flat panel detector, the XRpad 4336 (PerkinElmer Inc., US), with an imaging area of 35 cm × 43 cm. The X-ray generator is a light weight integrated system Transportix (Radiologia, Algete, Spain)) with 4 KW, 125 kVp, 100 mA, 0.001–10 s, 0.1–250 mAs, and dual focal spot 0.6–1.5 mm. The distance from source to detector is 125 cm, with a useful FOV for reconstruction of 17 cm.

**Fig 12:**
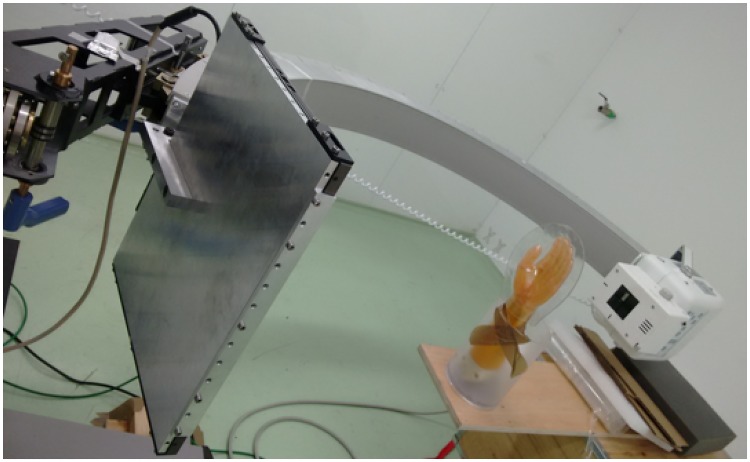
Setting with the in-house C-arm for the clinical phantom acquisition.

We obtained 49 projections, with a matrix size of 444×540 and pixel size of 0.8 mm, within an angular span of 120 degrees using the rotation along C-arm plane of both hand and foot, as shown in Fig 12.

**Fig 13:**
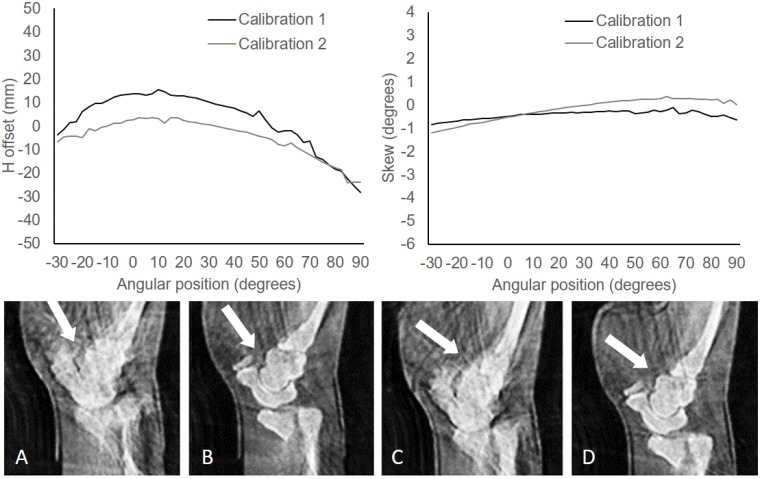
Top: Horizontal offset (left) and skew (right) for two calibrations. Bottom: Coronal views of the reconstructed image using FDK before (A, C) and after correcting the calibration parameters (B, D).

**Fig 14:**
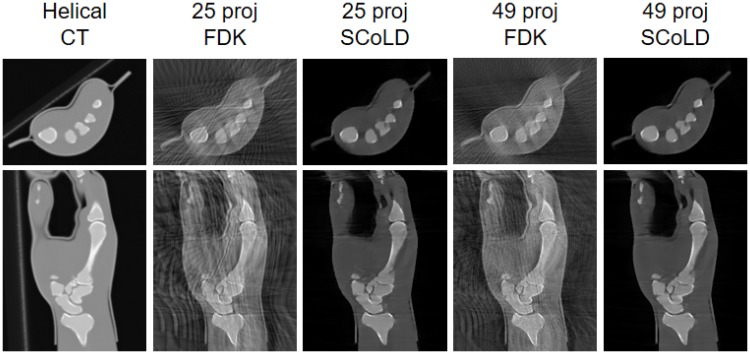
Axial and coronal views of the CT volume acquired on the helical scanner and of the reconstruction of the limited data obtained on the C-arm with an angular span of 120 degrees and 25 and 49 projections with FDK and SCoLD.

Fig 13 shows values of horizontal offset and skew of the detector for two calibrations obtained consecutively. The difference between the curves clearly demonstrates the non-repeatability of the source and detector positions and the need for a calibration refinement procedure. Reconstructions using FDK based on any single system calibration show severe artifacts (white arrows in Fig 14). The application of our calibration refinement algorithm to refine geometrical parameters *H*_*corr*_, *V*_*corr*_, and *S*_*corr*_ leads to a much-improved reconstruction (panels B and D).

Fig 14 shows the reconstructed volume of the hand using the refined geometrical calibration with FDK and SCoLD with the mask, for limited data with different number of projections. Reconstruction of a volume of 250×250×500 voxels (0.6 mm isotropic) took 5 and 6 minutes for 25 and 49 projections respectively.

We evaluated a possible implementation of the surface extraction using a *3D Artec Eva* scanner (Artec3D, Luxembourg), with a maximum spatial resolution of 0.5 mm and a 3D point accuracy of 0.1 mm, to scan the surface of the foot. The software *Artec Studio* was used to create a polygonal mesh from which a 3D binary mask of the surface was obtained. We could not replicate this experiment with the hand phantom since it is wrapped in a plastic cover that prevents the acquisition of its real surface with a surface scanner, as it can be seen in Fig 10 (top-right).

**Fig 15:**
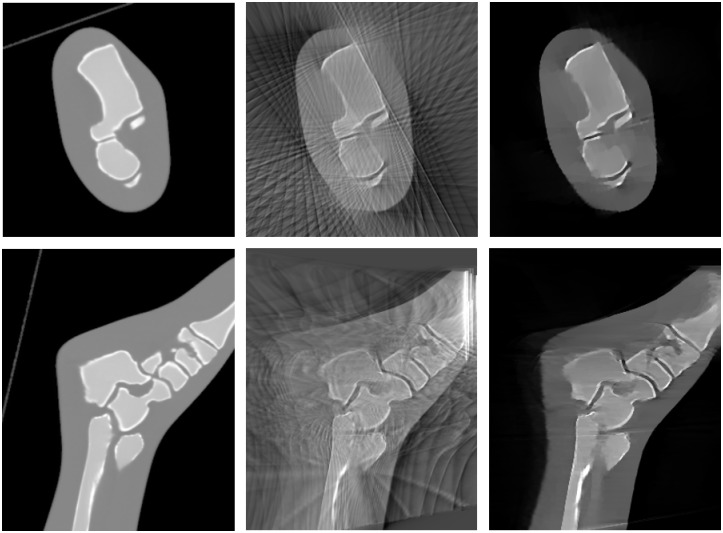
Axial and coronal views of the CT volume acquired on the helical scanner (left) and of the reconstruction of the limited data with 25 projections obtained on the C-arm with FDK (center), and with SCoLD using the acquired mask (right).

Fig 15 shows the result of the reconstructed image using the refined geometrical calibration with FDK and SCoLD using the mask obtained with the surface scanner. Reconstruction of a volume of 512×512×364 voxels (0.6 mm isotropic) took 15 minutes.

SCoLD presented significantly lower SAI and LiVA than FDK for the three cases, with an average reduction of 81% and 67.44% respectively (see Table 1).

**Table 1:** SAI and LiVA values for all cases.

	SAI		LiVA	
Phantom	FDK	SCoLD	FDK	SCoLD
Hand, 25 proj	130.97	26.61	55.29	18.89
Hand, 49 proj	136.91	27.73	49.89	18.90
Foot, 25 proj	209.60	32.87	62.54	16.02

## Discussion and conclusion

We propose a novel method to achieve tomographic capabilities using basic non-motorized C-arms, intended for planar imaging. There are three main challenges to address: (1) the trajectory of the source-detector pair may differ from a circular path and the system may suffer mechanical strains that modify the relative positions of the source and detector for different projection angles; (2) the exact position of the source and detector elements may not be repeatable for consecutive rotations due to low mechanical precision, thus preventing an accurate geometrical calibration of the system, and (3) the limitation of the angular span and the relatively low number of projections pose a great challenge to the image reconstruction, that leads to severe artifacts when using conventional reconstruction methods. The method proposed here is based on exploiting the surface information of the sample, which can be obtained with a 3D surface scanner. The results using data from two real C-arm systems, based on IR and flat panel detectors respectively, showed the feasibility of the proposal. The use of the surface of the sample enables the fine-tuning of geometrical calibration parameters and the removal of the artifacts derived from a low number of projections and limited angular span.

To address the issue of system calibration, Cho et al. [13] proposed a method specifically designed to obtain calibration parameters of a cone-beam system individually for each projection. However, this approach does not provide a complete estimation of the inclination angles (pitch and roll) because it is based on solving an optimization problem under the assumption that one of them is always different to zero. In the case of planar systems, not designed for obtaining tomographic data, this assumption may lead to artifacts, and for this reason we have extended the method by Cho et al. to include the estimation of the two inclination angles of the detector with respect to the horizontal and vertical directions independently.

Regarding the mechanical inaccuracy, which prevents a precise repetition of source-detector position between acquisitions, our results show that a periodic system calibration, standard in CT systems, is not enough to avoid misalignment artifacts in the reconstructed image. The *adaptive geometrical calibration* proposed here enables the refinement of three geometrical parameters, horizontal offset, vertical offset and skew, for the specific acquisition. Although possible errors in the rest of the geometrical parameters are neglected, results showed that this refinement increased accuracy so as to obtain images free of misalignment artifacts. The first step of the refinement algorithm used mutual information [22] to co-register the surface with the preliminary FDK reconstruction of the limited data. This might be difficult if the preliminary reconstructed volume is severely distorted due to a very low angular span or to truncation artifacts if the sample falls out of the field of view. This problem could be tackled by using markers on the sample, visible both in the acquired projection data and the surface scanner, which would allow a point-based registration instead. Given the high spatial resolution of the ARTEC EVA scanner used, radiopaque markers commonly used in the clinical practice (around 2 mm) could be used for this step.

Regarding the reduced angular span and the difficulty of obtaining a high number of projections, our *surface-constrained image reconstruction* reduces significantly the streak artifacts derived from the low number of projections, similarly to previous proposals in the literature [23–25]. However, its main advantage over previous works is that the restriction on the search space by exploiting the surface-based support results in a complete recovery of the external contour of the sample and adjacent areas even for extremely reduced angular spans. The reconstructed images showed an average SAI reduction of 81%, and limited angular span, with an average LiVA reduction of 67.44% when using SCoLD algorithm. Some horizontal patterns visible in the coronal views (Fig 15) can be explained by the fact that the present implementation of SCoLD calculates the derivatives only in 2D, as it can be seen in Eqs (13)–(16). We are currently implementing the 3D version, which we expect to avoid this effect.

The nominal accuracy of the ARTEC EVA scanner (0.1 mm) proved to be enough for the generation of the mask, since it is under the voxel size. However, the structured-light scanner showed two limitations: its elevated cost and possible errors due to the body hair or to the use of baggy clothes that may provide a wrong surface of the patient. The first problem could be solved using less expensive instrumentation, such as the Kinect camera system, and the second problem using infrared cameras.

The reconstruction time of several minutes may limit the use of the proposed method in some applications, like image-guided surgery where quasi real time is required. Optimization of this reconstruction time was out of the scope of this work, since our goal was to develop a proof of concept. Anyhow, we expect to reduce reconstruction times down to 50% by using high-performance workstations with multi-GPU architecture, as we have previously shown for similar algorithms [15]. Besides, a dedicated implementation for specific acquisition protocols would further reduce computational burden.

Evaluation was done with high contrast phantoms (soft tissue and bone). Further evaluation on more complex studies with more subtle soft-tissue contrast (abdomen) or tissue heterogeneity (chest) are advisable.

The use of the proposed method with other X-ray systems originally designed for planar imaging is straightforward provided that they allow moving the X-ray source and/or detector, opening the possibility of obtaining 3D information in other conventional radiology scenarios, such as tomosynthesis.
